# Long Noncoding RNA *H19* Mediates STAT3‐Dependent Activation of Keratinocytes and Fibroblasts in Systemic Sclerosis Skin

**DOI:** 10.1002/art.70104

**Published:** 2026-04-10

**Authors:** Begoña Caballero‐Ruiz, Christopher W. Wasson, Rebecca L. Ross, Francesco Del Galdo

**Affiliations:** ^1^ Leeds Institute of Rheumatic and Musculoskeletal Medicine University of Leeds Leeds United Kingdom; ^2^ NIHR Leeds Biomedical Research Centre, Leeds Teaching Hospitals, NHS Trust, Chapel Allerton Hospital Leeds United Kingdom

## Abstract

**Objective:**

Dermal systemic sclerosis (SSc) fibroblasts and their exosomes can activate keratinocytes in SSc, with long noncoding RNA (lncRNA) *H19* highlighted as the most up‐regulated RNA in their cargo compared with healthy controls (HCs). The role of *H19* in SSc pathogenesis has never been investigated. Here we determine *H19*'s role in the profibrotic activation of dermal fibroblasts and in their crosstalk with keratinocytes.

**Methods:**

Skin biopsies were obtained from the forearms of patients with diffuse SSc (n = 20) or HCs (n = 8), and dermal fibroblasts were explanted. In situ hybridization was used to determine *H19* expression in the skin, paired with immunohistochemistry of STAT3 and Collagen1. *H19* and STAT3 expression were modulated by retroviral transduction and small interfering RNA. Gel contraction and Transwell coculture were employed for functional studies.

**Results:**

In SSc, high *H19* expression in skin and explanted dermal fibroblasts compared with HCs showed moderate correlation to well‐described profibrotic markers. Knockdown of *H19* in SSc fibroblasts decreased profibrotic gene expression and cell contraction ability. *H19* overexpression was sufficient to induce a profibrotic phenotype in HC fibroblasts. Mechanistically, transforming growth factor‐β, interleukin‐6, and interleukin‐11 significantly increased *H19* expression in HC fibroblasts (2.2‐, 1.4‐, and 3.5‐fold, respectively) through activation of STAT3. Additionally, the STAT3 and STAT1 activation of keratinocytes induced by coculture with SSc fibroblasts was suppressed by *H19* knockdown.

**Conclusion:**

This study shows that lncRNA *H19* is a key mediator of STAT3‐induced changes in fibrotic activation of fibroblast and keratinocytes in SSc skin and could be considered a promising therapeutic target in tissue fibrosis.

## INTRODUCTION

Systemic sclerosis (SSc) is an autoimmune disease characterized by impaired angiogenesis and fibrosis of the skin and internal organs.^1^ Specific treatments for fibrosis in SSc, and other fibrotic diseases, are limited. Fibroblast to myofibroblast differentiation is well characterized as a key cellular event in tissue fibrosis. The transforming growth factor‐β (TGF‐β) pathway is one of the main signaling pathways known to induce myofibroblast activation through targeting transcriptional changes.^2^ However, beyond fibroblast activation there is a complex and dysregulated interplay of immune cells, endothelial cells, and epithelial cells that contribute to fibrosis. In the skin, keratinocytes are known to participate in tissue homeostasis via crosstalk with fibroblast and immune cells, which is found dysregulated in SSc.^3^ Intercellular communication is a crucial element of tissue homeostasis, and it is orchestrated both by soluble factors and extracellular vesicles better known as exosomes. The role of specific exosome cargoes in the aberrant crosstalk associated with tissue fibrosis in SSc is poorly characterized. Recently, we have shown that fibroblast exosomes can directly influence the pro‐inflammatory activation of keratinocytes and play a role in the innate immune activation observed in SSc skin.^4^ The analysis of SSc fibroblast exosome contents revealed long noncoding RNA (lncRNA) *H19* as one of the most significant differentially expressed genes in SSc dermal fibroblast derived exosomes compared with healthy controls (HCs).^4^

LncRNAs are defined as RNAs longer than 200 nucleotides that do not code for proteins.^5^ LncRNAs have been shown to have roles in modulating gene expression with aberrant expression linked to cancer and fibrosis. In SSc, some lncRNAs are known to be involved in the profibrotic activation of fibroblasts, including *HOTAIR* and MIR503, also known as *H19* located on the X chromosome.^6^, ^7^, ^8^, ^9^, ^10^, ^11^

An alternative lncRNA known as *H19*, an imprinted and maternally expressed 2.3‐kb lncRNA located on chromosome 11, is expressed in many tissues during embryo development and nearly completely down‐regulated in all tissues after birth, apart from the skeletal muscle.^12^ Recently, numerous studies showed *H19* to be involved in tumorigenesis and as a potential novel therapeutic target for fibrosis of the heart, liver, lungs, and kidney.^12^, ^13^, ^14^, ^15^ In addition, high levels of *H19* have been found in serum from patients with SSc^16^; however its role in regulating skin fibrosis in SSc is still unknown.

Here we show for the first time that *H19* levels are elevated in SSc skin and dermal fibroblasts ex vivo. Through gain and loss of function approaches we characterize the function of *H19* in dermal fibroblasts and its role in keratinocyte/fibroblast cell‐to‐cell communication.

## MATERIALS AND METHODS

### Patient enrollment, clinical characterization, and skin biopsies

Skin biopsies were obtained from the forearms of adult patients with diffuse SSc (n = 20) or HCs (n = 8) as control. All patients were male or female aged 22 to 78 years inclusive at screening with documented diagnosis of SSc, as defined using the 2013 American College of Rheumatology/EULAR criteria.^17^ All patients had the diffuse cutaneous form of SSc according to LeRoy and Medsger's criteria^18^; disease duration ≤3 years from the first non‐Raynaud phenomenon manifestation; a modified Rodnan skin score of 15 to 45 units at screening; and clinical skin involvement proximal and distal to the elbows, knees, or both or any truncal involvement, with or without face involvement. All participants provided written informed consent according to a protocol approved by the Medicine and Health Regulatory agency (NRES‐011NE to FDG, IRAS 15/NE/0211). Fibroblasts were isolated and established as previously described.^19^ Primary parental fibroblasts were immortalized using human telomerase reverse transcriptase (hTERT) to produce hTERT parental HC and diffuse SSc fibroblasts as previously described.^19^, ^20^ The demographic and clinical features of patients with diffuse SSc are summarized in Supplementary Table 1.

### Cell culture

Primary fibroblasts and human immortalized epidermal keratinocytes (HaCaTs; ATCC) were maintained in Dulbecco's modified Eagle medium (DMEM; Gibco) supplemented with 10% fetal bovine serum (FBS; Sigma) and penicillin‐streptomycin (Sigma) in a humidified incubator at 37°C and 5% CO_2_. For hTERT fibroblasts (HC n = 3; SSc n = 3), 250 μg/mL Geneticin (Thermo Fisher Scientific) was added into the medium. For assays, hTERT fibroblasts were cultured in medium without geneticin and used before passage 10. Primary fibroblasts were used before passage 4. Cells were routinely checked for mycoplasma.

### Morphogen stimulation

Cells were serum starved for 24 hours in DMEM containing 0.5% FBS prior treatment with SD208 inhibitor (1 μM, Sigma), cryptotanshinone (CTS; 5 μM, MedChemExpress), or DMSO as control for the time indicated in the figures. When specified, inhibitor cells were stimulated with 10 ng/mL TGF‐β (R&D Systems), 50 ng/mL interleukin (IL) 6 (PeproTech) or 50 ng/mL IL‐11 (PeproTech) in a humidified incubator at 37°C and 5% CO_2_.

### Quantitative polymerase chain reaction

Total RNA was isolated using commercial RNA extraction kits (Zymo Research) and quantified by A260nm/A280nm in a Nanodrop. Synthesis of complementary DNA (cDNA) was performed using 500‐ng RNA with cDNA synthesis kits (Thermo Fisher). Real‐time quantitative polymerase chain reaction (PCR) was performed using 1 μL of 10 ng/μL cDNA and target‐specific primers *H19* (forward: 5′‐TCAAGCCTGGGCCTTTGAAT; reverse: 5′‐GGCTGATGAGGTCTGGTTCC), *STAT3* (forward: 5′‐TCTGTGTGACACCAACGACC; reverse: 5′‐TCCTCACATGGGGGAGGTAG), *Collagen type 1A1* (*Col1A1*; forward: 5′‐ CCTCCAGGGCTCCAACGAG; reverse: 5′‐ TCTATCACTGTCTTGCCCCA), α‐*ACTA2* (forward: 5′‐TGTATGTGGCTATCCAGGCG; reverse:5′‐ AGAGTCCAGCACGATGCCAG), *CCN2* (forward: 5′‐CTTGCGAAGCTGACCTGGAAGA; reverse: 5′‐CCGTCGGTACATACTCCACAGA), and *GAPDH* (forward: 5′‐ ACCCACTCCTCCACCTTTGA; reverse: 5′‐ CTGTTGCTGTAGCCAAATTCGT). A SyBr Green PCR kit with a CFX Connect Real‐Time PCR Detection System (Bio‐Rad) thermocycler was used. Amplification was quantified and expressed following the ΔΔCt method using *GAPDH* as a housekeeping gene.

### Western blotting

Total protein was extracted in a radioimmunoprecipitation assay buffer with proteinase and phosphatase inhibitors (Sigma) and resolved by sodium dodecyl sulfate‐polyacrylamide gel electrophoresis, transferred onto nitrocellulose membranes (Amersham Biosciences), and probed with antibodies specific for GAPDH (Proteintech, HRP‐60004), pSTAT3 Tyr 705 (Cell Signaling, 9131), STAT3 (Abcam, 119352), connective tissue growth factor (CTGF/CCN2; Abcam, ab209780), pSMAD3 (Abcam, ab52903), SMAD3 (Cell Signaling, 9523), pSTAT1 (Cell Signaling, 9167), and STAT1 Tyr 701 (Cell Signaling, 9172). Immunoblots were visualized with species‐specific HRP‐conjugated secondary antibodies (Sigma) and Supersignal West Pico PLUS Chemiluminescent Substrate (Thermo Fisher, Cat. No. 34577) on a Bio‐Rad chemiDoc imaging system using the Image Lab software (Bio‐Rad) according to the manufacturer's instructions. Signal intensity was quantified by ImageJ (Fiji) and normalized to the loading control indicated in the text.

### RNAscope and costaining

For in situ hybridization of *H19* in skin biopsies we used the RNAscope probe designed by Biotechnne (Cat. No. 1152841‐C1) together with the RNAscope 2.5HD Detection kit RED (Cat. No. 322360) following the manufacturer's instructions. The probe was hybridized for 2 hours at 40°C using the HybEZ Oven provided. Signal detection was obtained using the Fast RED A/B solution provided in the kit. We used 50% Hematoxylin Gill NO.1 (Sigma) to counterstain the slides before mounting.

For codetection of *H19* with Collagen1 (1:100, Abcam 6308) or STAT3 (1:100, Abcam 119352) we used the RNA‐Protein codetection Ancillary kit from Biotechnne (Cat. No. 323180), which enables combination of RNAscope with immunohistochemistry‐compatible antibodies. Primary antibody was applied overnight (ON) at 4°C. The day after, 10% neutral buffered formalin was added for 30 minutes at room temperature for postprimary fixation. Then we applied the corresponding RNAscope protease for 30 minutes at 40°C. Final detection was performed using 3, 3 ‐diaminobenzidine (Vector Laboratories) and counterstaining with 50% Hematoxylin Gill NO.1 (Sigma). Slides were scanned using a Leica Biosystems AT2 digital slide scanner at 20× resolution and Zeiss AxioImager.Z1 microscope using 40× resolution.

RNAscope and costaining quantification was performed using ImageJ and represented as the percentage of the probe per area (μm^2^) on the indicated samples per group. Four images per sample were used for quantification in the epidermis and subepidermal regions.

### Small RNA interference transfections

Two gene‐specific 27mer small interfering RNA (siRNA) duplexes specific for *H19* (siRNA 1 and siRNA2) or a scramble (SCR) negative control siRNA duplex from Origene (Cat.No. SR319206) were transfected into SSc hTERT fibroblasts using siTrans 2.0 (Cat. No. TT320001) in complete medium (10% FBS). After 5 hours, fresh medium was added. Fibroblasts were incubated for the indicated times in complete or starved media (0.5% FBS) before harvesting.

A unique siRNA specific for *STAT3* or SCR negative control sequence from Qiagen (Cat. No. SI00048363) was transfected into fibroblasts using Lipofectamine 2000 (Thermo Fisher). After 24 hours, we medium‐changed the cells with complete or starved media for the indicated times before harvesting.

### Micro‐RNA29a transfection

Fibroblasts were transfected in antibiotic‐free media with 10 nM of micro‐RNA 29a (miRNA29a or miR29a) miRIDIAN miRNA mimic or negative control miRNA (Horizon) using the DharmaFECT4 (Horizon, Cat. No. T‐2004‐02) transfection reagent. Fibroblasts were then incubated for 48 hours before harvesting.

### Retroviral transduction

*H19* expression was induced by transduction with retroviruses carrying pBabepuro vector containing the human *H19* sequence (NR_002196.3) or SCR sequence as control (Dundee University, UK). For this purpose, hTERT healthy dermal fibroblasts were seeded 50% confluence and infected with retroviral particles in full media ON. The day after cells were medium‐changed with full media before a second retroviral transduction ON. Transduced fibroblasts were maintained in full media for 48 hours then selected with 1.0 μg/mL Puromycin (Gibco) for another 48 hours. Cells were maintained in 0.5 μg/mL Puromycin and 250 μg/mL Geneticin (Thermo Fisher Scientific).

### Gel contraction

On the day of the assay hTERT fibroblasts were trypsinized and seeded at 200,000 cells on gel/cell mixture per well of a 24‐well plate. Briefly, the Collagen Gel Working Solution (Cambridge Biosiences, Cat.No. CBA‐201) was prepared according to the manufacturer's instructions in a cold sterile tube. The desired number of cells was resuspended in low‐glucose DMEM 1% FBS and 1% penicillin‐streptomycin in the volume needed to prepare one part of cell mixture and four parts of cold Collagen Gel Working Solution. A total of 0.5 mL of cell collagen mixture was added per well and incubated 1 hour at 37°C in an incubator without CO_2_. After collagen polymerization, 1 mL of 1% FBS culture medium was added on top of each collagen gel and incubated ON at 37°C and 5% CO_2_. The next day, the sides of the cell collagen gels were detached using sterile spatulas ensuring that the gels were circular. Pictures of the gels were taken at the indicated time points and weight at the last time point. For analysis, the scale was set using the known distance of the bottom of a 24 well plate, and the area of each gel was measured at various time points using ImageJ (Fiji).

### Chromatin immunoprecipitation analysis

Chromatin immunoprecipitation (ChIP) was performed according to the protocol published by Thermo Fisher with some modifications. Briefly, cells were harvested and resuspended in 1% paraformaldehyde for 15 minutes to induce crosslinking. Formaldehyde was quenched with 1.25 M glycine. Then, the cells were lyzed in a two‐step process with cellular (50 mM Tris‐HCl pH 7.5, 1 mM EDTA pH 8.0, 140 mM NaCl, and 1% NP‐40) and nuclear lysis buffer (50 mM Tris‐HCl pH 7.5, 2 mM EDTA pH 8.0, 0.5% sodium deoxycholate, and 1% sodium dodecyl sulfate [SDS]). The DNA was sheared by sonication (2 × 30 seconds 50 mHz). The sheared chromatin was incubated with pSTAT3 Tyr 705 (Cell Signaling, 9131) or IgG (Thermo Fisher, Cat No. 86199) control antibody for 2 hours at room temperature. Then, Dynabeads Protein G (Thermo Fisher, Cat No. 10004D) were added to the mixture ON at 4°C. The beads were then washed in low‐salt (20 mM Tris‐HCl pH 8.0, 2 mM EDTA pH 8.0, 0.1% SDS, 1% Triton X‐100, and 150 mM NaCl) and high‐salt (20 mM Tris‐HCl pH 8.0, 2 mM EDTA pH 8.0, 0.1% SDS, 1% Triton X‐100, and 500 mM NaCl) wash buffers, and then the DNA was eluted with elution buffer (1% SDS, 100mM NaHCO_3_). Crosslinking was reversed with 5 M NaCl for 3 hours at 65°C. The DNA was treated with RNase (Thermo Fisher) and then Proteinase K (Macherey‐Nagel). DNA was then purified using a DNA clean‐up kit (Qiagen, Cat No. 28104). Co‐immunoprecipitated DNA was detected by quantitative PCR using primers to *H19* promoter (forward: 5′‐ATGTGGCTCCCATGAATGTC; reverse: 5′‐GGCTCTTGCATAGCACATGA) and primers to β‐catenin promoter as positive control (forward: 5′‐AATTGGAGGCTGCTTAATCG; reverse: 5′‐TTCCATTTTTATCTGGTTCCAC). The data were normalized according to the percent input method.

### Transwell assay

For coculturing assays, on day 1, 90% HC and/or SSc hTERT fibroblasts were seeded per well in six‐well plates, and 90% HaCaTs were seeded separately per 0.40‐μm pore translucent high‐density polyethylene terephthalate membrane (Corning) in full media. For assays with knockdown cells, on day 2, SSc hTERT dermal fibroblasts were transfected with *H19* siRNA as previously described. On day 3, cells reached 100% confluency and insert and wells were washed with phosphate‐buffered serum. The inserts containing HaCaTs were added on top of each well containing fibroblasts, and serum‐free media was added. After 48 hours, cells were harvested for RNA and protein.

### Statistical analysis

GraphPad Prism 10 was used for statistical analysis and for the generation of graphs. Results were presented as mean and SEM. One‐way analysis of variance parametric test or Student's *t*‐test were performed for experiments, with *P* < 0.05 considered significant. All data used in this research are already included in the article or the Supplementary Materials. Further information is available from the corresponding author upon reasonable request.

## RESULTS

### LncRNA *H19* is up‐regulated in SSc skin and explanted dermal fibroblasts and correlates with key markers of fibrosis

**Figure 1:**
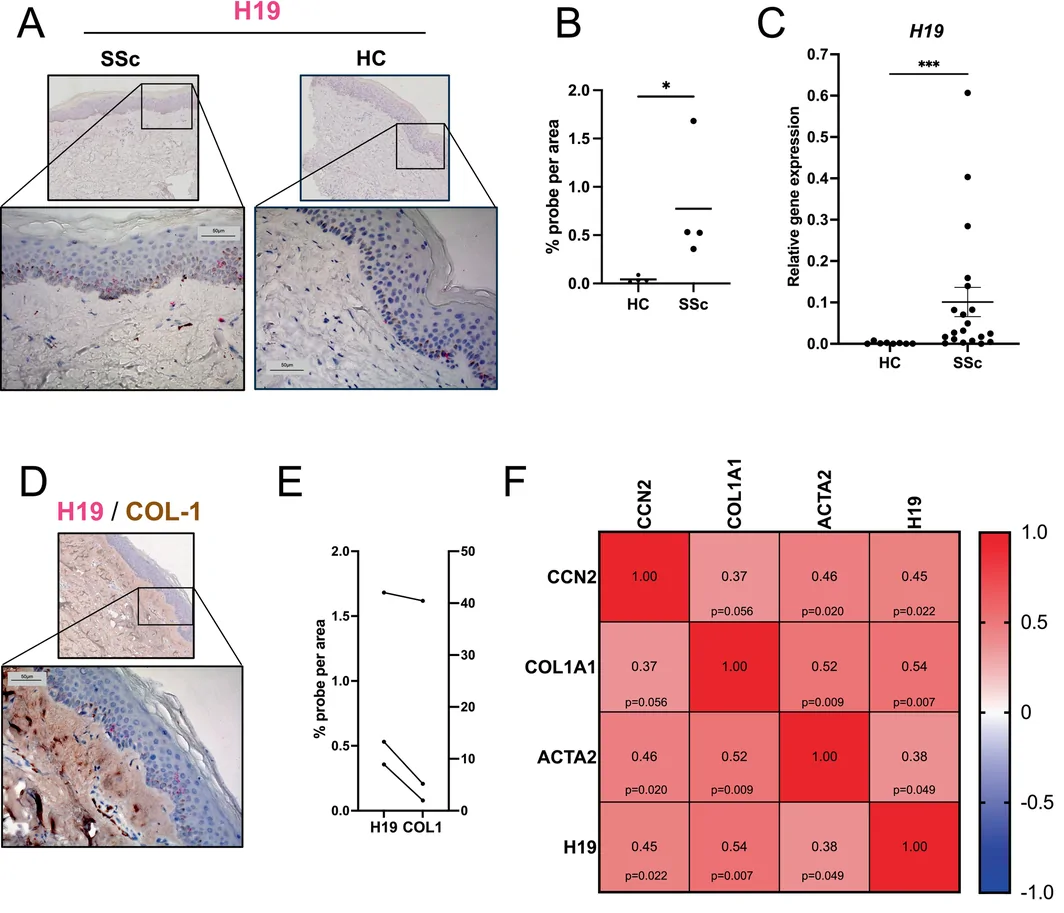
Increased *H19* in SSc skin and explanted dermal fibroblast correlates to profibrotic markers. (A) RNAscope of *H19* (pink) in skin samples. Representative images of one HC and one SSc are shown. Top image represents low‐resolution insert and the bottom the high resolution of the selected area. Scale bar = 50 μm. (B) Graph represents the percentage of *H19* probe per area (μm^2^) (n = 4 per group). (C) mRNA transcript levels of *H19* normalized to *GAPDH* mRNA levels in primary HC (n = 8) and SSc fibroblast (n = 20). Data show mean ± SEM, unpaired *t*‐test (*P* < 0.001). (D) RNAScope of *H19* (pink) co‐stained with COL‐1 (brown) in representative SSc skin sample with high *H19* levels. Scale bar = 50 μm. (E) The graph represents the percentage of *H19* and COL1 costaining per sample (n = 3). (F) Correlation matrix of *H19* with mRNA levels of the indicated profibrotic markers in 20 SSc primary fibroblast. Values represent Spearman *r* correlation coefficient. Red represents increased expression, and blue represents decreased expression. *P < 0.05; ***P < 0.001. COL‐1, Collagen 1; HC, healthy control; mRNA, messenger RNA; SSc, systemic sclerosis.

Dermal SSc fibroblasts have been shown to have increased *H19* in their secreted exosomes compared with HCs,^4^ thus we wanted to assess whether differences in *H19* levels could be observed within the whole skin. RNAscope (RNA in situ hybridization) targeting *H19* in skin samples indicated higher *H19* levels in SSc skin (n = 4) compared with HCs (n = 4) (18.2‐fold change in the percentage of *H19* probe per area [μm^2^]; *P* < 0.05), with increased levels of *H19* being detectable in the epidermal layer as well as within the dermis and the adipose tissue of SSc skin (Figure 1A and B; Supplementary Figure 1A).

Ex vivo, SSc explanted dermal fibroblasts showed markedly increased expression of *H19* (59.6‐fold change; *P* < 0.001) compared with fibroblasts isolated from HC skin (reverse transcription–quantitative PCR [RT‐qPCR] analysis; SSc n = 20; HC n = 8) (Figure 1C). Similar to the results in skin biopsies, we observed high variability of *H19* expression within SSc fibroblast lines, which also was maintained in vitro following hTERT‐mediated immortalization (Supplementary Figure 1B).

Immunohistochemistry studies paired with in situ hybridization revealed that the heterogeneity in *H19* expression aligns with the amount of Collagen 1 (COL‐1) profibrotic marker expressed in SSc skin. Despite the small size number, SSc skin tissue with the highest *H19* expression showed the highest collagen staining and vice versa (Figure 1D and Supplementary Figure 1C represent skin levels of *H19* [pink] and COL‐1 within the dermis [brown]; Figure 1E represents the percentage of *H19* and COL‐1 per sample, n = 3). Consistent with this observation, RT‐qPCR studies of explanted SSc fibroblasts in vitro showed a moderate, albeit significant, correlation between *H19* levels and messenger RNA (mRNA) levels of *Col1A1* (*R* = 0.54, *P* = 0.007), α‐smooth muscle actin (encoded by *ACTA2*) (*R* = 0.38, *P* = 0.049), and *CTGF* (also known as *CCN2*) (*R* = 0.45, *P* = 0.022) (Figure 1F). Altogether, these findings supported a role of *H19* in the context of profibrotic activation of dermal fibroblasts in SSc.

### *H19* expression regulates profibrotic activation of dermal fibroblasts in vitro

**Figure 2:**
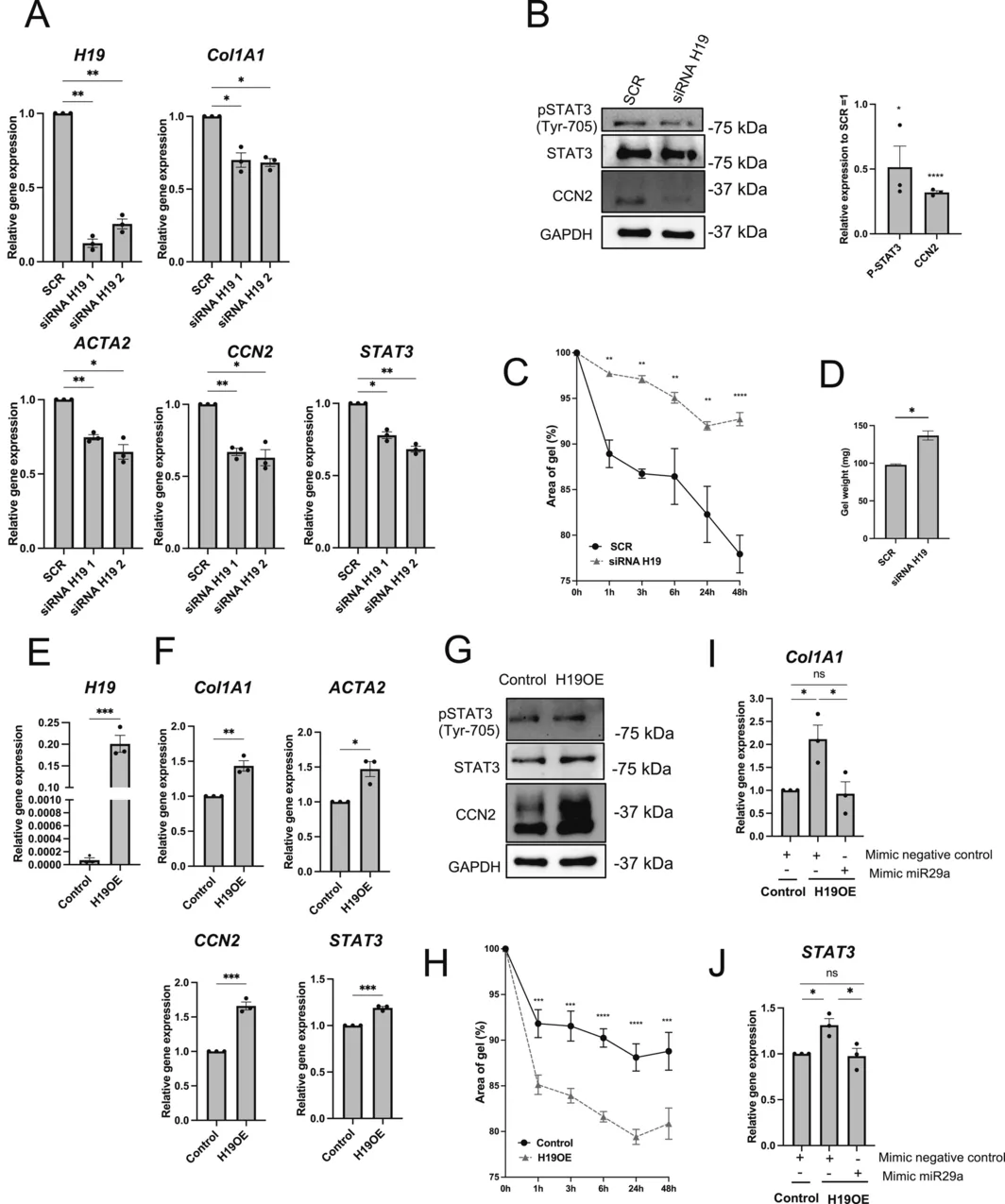
*H19* knockdown decreased profibrotic activation of SSc fibroblasts, in vitro. On the contrary, overexpression of *H19* induced profibrotic activation of HC dermal fibroblasts. (A) mRNA expression in SSc SCR and *H19* knockdown fibroblast using siRNA1 and 2 at 48 hours in serum‐starved media. Normalized to *GAPDH* mRNA levels, fold change to SCR. (B) Left: Western blot of the indicated markers in SSc fibroblast transfected with SCR and *H19* siRNA1 at 48 hours in serum‐starved media. Right: densitometry of indicated markers relative to SCR (SCR = 1). (C) Area of collagen gel in percentage over time of SSc SCR (black) and *H19* knockdown fibroblast using siRNA1 (gray). (D) Gel weight in milligrams after 48 hours of the gel conditions shown in panel C. (E) *H19* mRNA expression normalized to *GAPDH* mRNA levels in HC fibroblast H19OE and control. (F) mRNA expression in HC fibroblast H19OE and control. Normalized to *GAPDH* mRNA levels, fold change to control. (G) Western blot of the indicated markers in HC H19OE and control. (H) Area of collagen gel in percentage over time of healthy fibroblast H19OE (gray) and control (black). (I) *Col1A1* mRNA expression in control and H19OE transfected with mimic negative control or mimic miR29a for 48 hours. Normalized to *GAPDH* mRNA levels, fold change to control. (J) *STAT3* mRNA expression from panel I. Data show mean ± SEM. *P < 0.05; **P < 0.01; ***P < 0.001; ****P < 0.0001. HC, healthy control; mRNA, messenger RNA; ns, not significant; SCR, scramble; siRNA, small interfering RNA; SSc, systemic sclerosis.

Next, we established loss and gain of function models to determine whether the increased expression of *H19* could play a direct role in profibrotic activation of dermal fibroblasts in SSc. To knockdown the expression of *H19* we employed two siRNAs, which achieved 88% and 74% knockdown (siRNAs H19 1 and 2, respectively) versus scrambled RNA (SCR) after 48 hours. RT‐qPCR analysis showed that knockdown of *H19* decreased the gene expression of profibrotic markers *Col1A1* (0.70‐fold, *P* < 0.05 and 0.68‐fold, *P* < 0.05), *ACTA2* (0.74‐fold, *P* < 0.01 and 0.62‐fold, *P* < 0.05), *CCN2* (0.66‐fold, *P* < 0.01 and 0.62‐fold, *P* < 0.05), and *STAT3* (0.78‐fold, *P* < 0.05 and 0.69‐fold, *P* < 0.01) relative to SCR SSc fibroblasts (Figure 2A). Western blot analysis showed that the knockdown of *H19* using siRNA1 partially suppressed protein levels of pSTAT3 (Tyr‐705) and CCN2 (Figure 2B).

SSc fibroblasts have previously been shown to have a higher ability to contract collagen gel compared with HC.^19^ SSc fibroblasts with reduced expression of *H19* using siRNA H19 1 showed a 3.1‐fold reduction in collagen gel contraction (7% vs 22% from SCR) after 48 hours (Figure 2C; Supplementary Figure 2A). On the other hand, siRNA H19 2 showed a 1.4‐fold reduction in collagen gel contraction (16% contraction vs 22% from SCR) (Supplementary Figure 2B). The difference in performance was associated with a similar difference in efficiency of maintaining reduced *H19* levels over time (Supplementary Figure 2C). Consistently, gel weight was significantly increased by 38% after 48 hours when in *H19‐*siRNA 1 fibroblasts (*P* < 0.05), supporting the observation of a lesser contraction of the matrix (Figure 2D).

In a complementary approach, we established *H19* overexpressed (H19OE) healthy dermal fibroblast (Figure 2E). Overexpression of *H19* induced an increase in gene expression of *Col1A1* (1.43‐fold, *P* < 0.01), *ACTA2* (1.47‐fold, *P* < 0.05), *CCN2* (1.66‐fold, *P* < 0.001), and *STAT3* (1.2‐fold, *P* < 0.001) relative to HC fibroblast (Figure 2F). Consistent with these findings, protein analysis showed a slight increase in STAT3 as well as an increase in CCN2 protein levels (Figure 2G). Functionally, *H19* overexpression significantly increased gel contraction ability of HC fibroblast by 1.7‐fold after 24 hours (*P* < 0.0001) (20.47% contraction vs 11.8% from HC) (Figure 2H; Supplementary Figure 2D).

*H19* is known to sponge miRNAs as one of its most studied regulatory mechanisms.^8^ Previously, *H19* has been described to sponge miR29a,^21^, ^22^ and miR29a is a key regulator of COL‐1 in SSc.^23^, ^24^ Interestingly, in cancer cells, miR29a has been shown to also target STAT3.^25^, ^26^ To better understand the regulation of these markers by *H19*, we transfected our H19OE and HC fibroblast with mimic miR29a and its relative negative control. Introduction of miR29a was able to reverse the ability of *H19* to induced *Col1A1* (*P* < 0.05) and *STAT3* (*P* < 0.05) levels (Figure 2I and J). Taken together, these results indicate that *H19* can have a direct role in the profibrotic phenotype of SSc fibroblasts, and inducing *H19* is sufficient to activate a strong profibrotic phenotype in HC fibroblasts.

### LncRNA *H19* is regulated by STAT3 in dermal fibroblasts

The regulation of *H19* has been explored in other cellular models, where previously it has been described downstream of SMADs and of STAT3.^27^, ^28^ Consistent with these models, Li et al have shown that TGF‐β can significantly induce *H19* levels in hepatic stellate cells in the context of profibrotic activation.^5^ The TGF‐β canonical signaling pathway signals via TGF‐β receptor type 1 mediating SMAD proteins. On the other hand, STAT3 is activated by several inflammatory cytokines, as IL‐6, and phosphorylated at Tyr‐705 by kinases such as JAK, SRC, and JNK, also mediators of the TGF‐β noncanonical pathways.^29^

**Figure 3:**
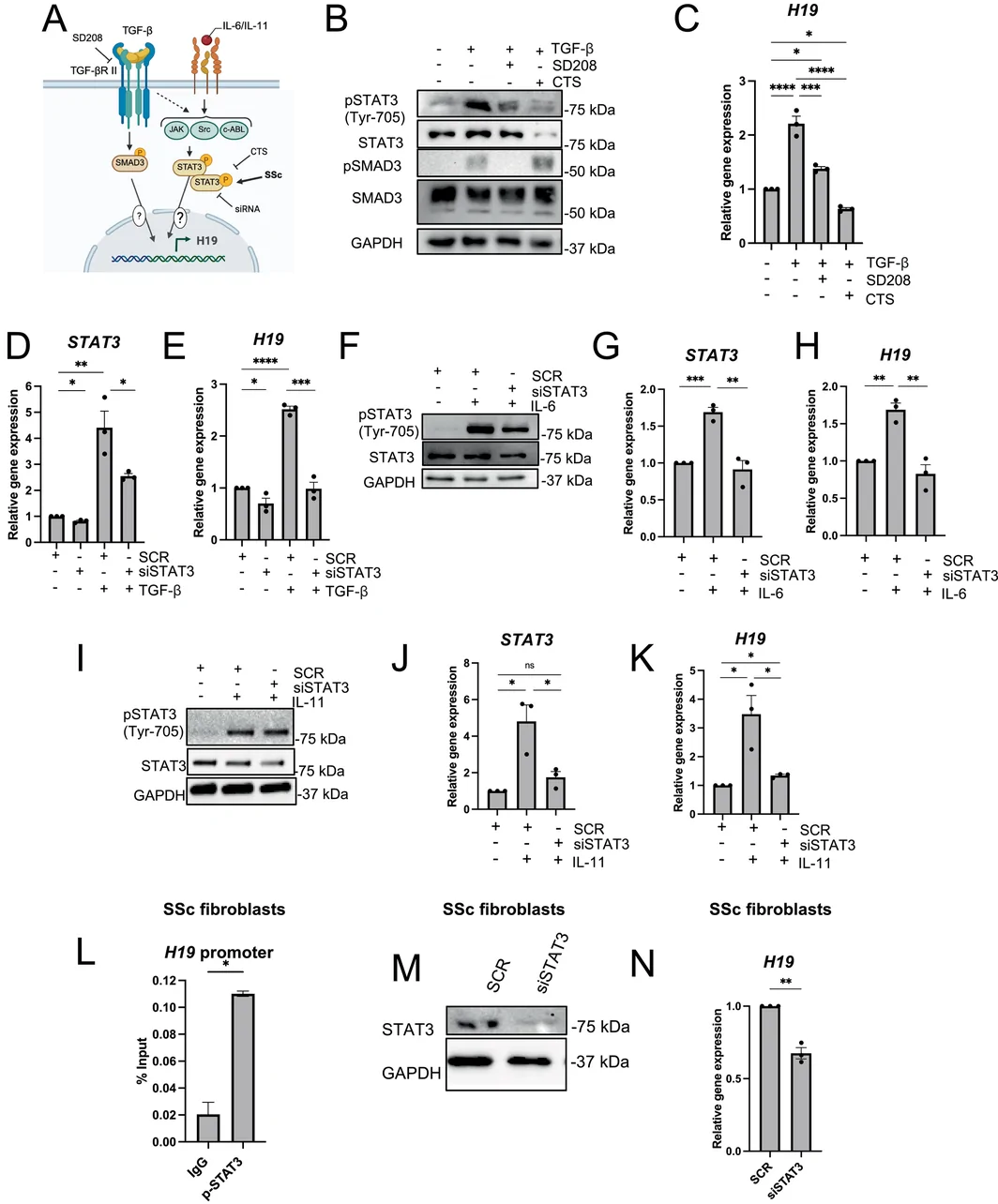
*H19* is regulated by STAT3 in dermal fibroblast. (A) Schematic design of the canonical and noncanonical TGF‐β pathway, IL‐6/IL‐11 pathways, and the different inhibitors used (created with BioRender.com). (B) Western blot of the indicated markers in HC fibroblast serum starved ON, followed by SD208 or CTS for 24 hours and another 24 hours of TGF‐β or the corresponding control. (C) *H19* mRNA levels normalized to *GAPDH* from panel B. (D) *STAT3* mRNA levels normalized to *GAPDH* in HC fibroblast transfected with siSTAT3 or SCR, 24‐hour later cells were serum starved ON and 24‐hour TGF‐β induced or corresponding control. (E) *H19* mRNA levels normalized to *GAPDH* from panel D. (F) Western blot of the indicated markers in HC fibroblast transfected with siSTAT3 or SCR, 24‐hour later cells were serum starved ON and 6‐hour IL‐6 or corresponding control. (G) *STAT3* mRNA levels normalized to *GAPDH* from panel F. (H) *H19* mRNA levels normalized to *GAPDH* from panel F. (I) Western blot of the indicated markers in HC fibroblast transfected with siRNA STAT3 or SCR, 24‐hour later cells were serum starved ON and 6‐hour IL‐11 or corresponding control. (J) *STAT3* mRNA levels normalized to *GAPDH* from panel I. (K) *H19* mRNA levels normalized to *GAPDH* from panel I. (L) Chromatin immunoprecipitation assay performed using p‐STAT3 antibody and IgG control, followed by reverse transcription–quantitative polymerase chain reaction using primers specific for *H19* promoter. The data were normalized according to the percentage input method. (M) Western blot of STAT3 and GAPDH in SSc fibroblast transfected with siSTAT3 for 24 hours and then serum‐starved media for another 48 hours. (N) *H19* mRNA levels normalized to *GAPDH* from panel M. Data shows mean ± SEM. *P < 0.05; **P < 0.01; ***P < 0.001; ****P < 0.0001. CTS, cryptotanshinone; HC, healthy control; IL, interleukin; mRNA, messenger RNA; ns, not significant; ON, overnight; SCR, scramble; siRNA, small interfering RNA; siSTAT3, STAT3 with specific siRNA; SSc, systemic sclerosis; TGF‐β, transforming growth factor‐β. Color figure can be viewed in the online issue, which is available at http://onlinelibrary.wiley.com/doi/10.1002/art.70104/abstract.

To explore the regulation of *H19* expression in dermal fibroblasts, our first approach was to stimulate HC fibroblast with TGF‐β and in combination with the TGF‐βR inhibitor (SD208) or STAT3 dimerization inhibitor (CTS) (Figure 3A). Protein analysis showed that TGF‐β induced phosphorylation of SMAD3 and STAT3 (Tyr‐705), which was suppressed by SD208, as expected. Treatment with CTS decreased total STAT3 levels and prevented its phosphorylation (Tyr‐705) while leaving SMAD3 phosphorylation levels unaffected (Figure 3B). In this experimental setting, TGF‐β stimulation significantly increased *H19* gene expression by 2.2‐fold in HC fibroblasts (*P* < 0.0001), which was reduced by SD208. On the contrary, prevention of STAT3 dimerization led to a significant decrease (71%; *P* < 0.0001) in *H19* levels (Figure 3C).

In a complementary approach, we induced *H19* expression in HC fibroblasts with TGF‐β and directly targeted STAT3 with specific siRNA (siSTAT3). STAT3 knockdown was assessed by mRNA levels (Figure 3D). Consistent with our previous findings, siRNA targeting of STAT3 significantly suppressed TGF‐β–mediated *H19* induction (*P* < 0.001) (Figure 3E). These results suggested that the effects of TGF‐β on *H19* are directly mediated by STAT3.

To explore the effect of other sources of STAT3 activation on *H19* levels, we treated HC fibroblast with human recombinant IL‐6. Six hours of IL‐6 stimulation induced a significant (46‐fold) phosphorylation of STAT3 (Tyr‐705) as well as an increase in *STAT3* mRNA levels by 1.7‐fold, as expected (Figure 3F and G). In this experimental setting, *H19* expression was induced by 1.7‐fold (*P* < 0.001), which was entirely suppressed by siRNA knockdown of STAT3 despite a limited efficiency (42%) (Figure 3H). RNA‐sequencing of primary human dermal fibroblast revealed that the IL‐11 receptor α subunit is one of the most highly expressed receptors part of the IL‐6 family.^30^ Consistently, 6 hours of IL‐11 stimulation also induced a significant phosphorylation of STAT3 (Tyr‐705) and *STAT3* mRNA levels by 4.8‐fold (*P* < 0.05) (Figure 3I and J). *H19* expression was induced by 3.5‐fold (*P* < 0.05), which was partially suppressed by siRNA knockdown of STAT3 (Figure 3K).

STAT3 activation is consistently reported to be increased in SSc skin and fibroblasts.^29^ Furthermore, a previous study showed that pSTAT3 can access the promoter of *H19* in pancreatic cancer cells.^31^ Here, using ChIP analysis, we showed that pSTAT3 binds to the *H19* promoter in SSc dermal fibroblasts. pSTAT3 was enriched five‐fold at the *H19* promoter compared with the IgG control (Figure 3L). This suggests a role for STAT3 in the regulation of *H19* expression in SSc dermal fibroblasts. This was confirmed when we knocked down STAT3 in SSc dermal fibroblasts, given their known increased STAT3 activation.^29^ Silencing of STAT3 in SSc dermal fibroblasts led to a significant suppression of baseline *H19* expression (*P* < 0.01) (Figure 3M and N). Taken together, these data suggest *H19* expression in fibroblasts is regulated by STAT3 both in HC fibroblasts following TGF‐β or IL‐6/IL‐11 stimulation and in SSc dermal fibroblasts.

### LncRNA *H19* dermal fibroblast expression can modulate cell‐cell communication and induction of STAT3 activation in epidermal cells

**Figure 4:**
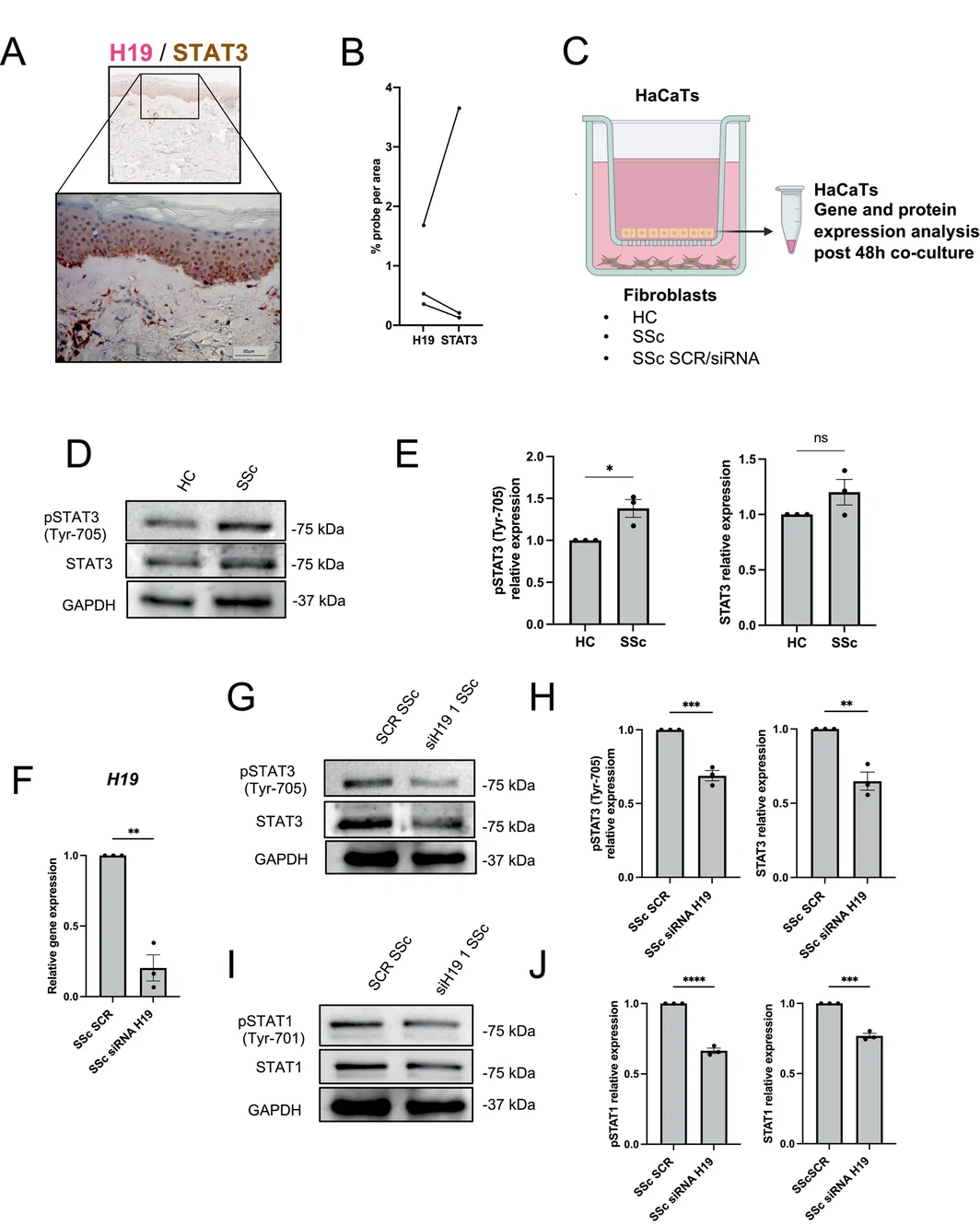
SSc fibroblast‐induced STAT3 and STAT1 activation in epidermal cells is dependent on *H19*. (A) RNAScope of *H19* (pink) co‐stained with STAT3 (brown) in representative SSc skin. Scale bar = 50 μm. (B) Graph represents the percentage of *H19* and STAT3 costaining per sample (n = 3). (C) Schematic design of the fibroblast and HaCaTs coculture. (D) Western blot of the indicated markers in HaCaTs cocultured with HC or SSc fibroblast for 48 hours. (E) Densitometry of pSTAT3 (Tyr‐705) protein expression (left) and total STAT3 (right) normalized to GAPDH from panel D. Represented as fold change to HC. (F) mRNA transcript levels of *H19* in SSc SCR or *H19* knockdowned after 48 hours of coculture and 72 hours after transfection. (G) Western blot of the indicated markers in HaCaTs cocultured for 48 hours with SSc SCR and *H19* knockdowned fibroblasts. (H) Densitometry of pSTAT3 (Tyr‐705) protein expression (left) and total STAT3 (right) normalized to GAPDH from panel G. Represented as fold change to SCR SSc. (I) Western blot of the indicated markers in HaCaTs cocultured for 48 hours with SSc SCR and *H19* knockdowned fibroblasts. (J) Densitometry of pSTAT1 protein expression (left) and total STAT1 (right) normalized to GAPDH from panel H. Represented as fold change to SCR SSc. Data show mean ± SEM. *P < 0.05; **P < 0.01; ***P < 0.001; ****P < 0.0001. HaCaT, human immortalized epidermal keratinocyte; HC, healthy control; mRNA, messenger RNA; ns, not significant; SCR, scramble; siRNA, small interfering RNA; SSc, systemic sclerosis. Color figure can be viewed in the online issue, which is available at http://onlinelibrary.wiley.com/doi/10.1002/art.70104/abstract.

We previously observed *H19* expression in the epidermis (Figure 1A) and STAT3 being both upstream and downstream of *H19* regulation in dermal fibroblasts (Figures 2 and 3). This is of interest as STAT3 signaling has been reported to regulate keratinocyte differentiation and skin homeostasis, which is disrupted in SSc.^32^, ^33^ We next wanted to assess the effect of *H19* in keratinocyte STAT3 activation. Costaining for *H19* and STAT3 in SSc skin suggested that higher levels of *H19* align to high STAT3 protein levels, colocalizing within the lower base of the epidermis and fibroblasts (representative images are shown in Figure 4A; Figure 4B shows the quantification of the percentage of STAT3 and *H19* per area). Because fibroblast to keratinocyte communication, partially via exosomes, is known to be key in skin homeostasis,^4^ we hypothesized that higher *H19* levels in SSc fibroblasts could play a key role in keratinocyte STAT3 activation. To investigate this, we first cocultured SSc and HC fibroblast with HaCaTs in which we assessed STAT3 levels after 48 hours (Figure 4C). SSc fibroblasts significantly induced phosphorylation of STAT3 (Tyr‐705) by 1.4‐fold (*P* < 0.05) in HaCaTs compared with HC fibroblast (Figure 4D and E). Next, we cocultured HaCaTs with SSc fibroblast with *H19* knockdown (siRNA1; Figure 4F). Importantly, knockdown of *H19* in SSc fibroblasts prevented keratinocyte activation as assessed by the total and phosphorylation protein levels of STAT3 (Figure 4G and H). Previously, our group showed that SSc fibroblasts can induce phosphorylation of STAT1 in keratinocytes when cocultured in vitro^34^ and through exosomes.^4^ Interestingly, *H19* knockdown in fibroblasts also prevented phosphorylation of STAT1 in HaCaTs (Figure 4I and J). All together, we conclude that *H19* expression in dermal fibroblasts can regulate STAT3 and STAT1 activation in epidermal cells.

## DISCUSSION

The importance of fibroblasts to keratinocyte crosstalk maintaining skin homeostasis in SSc has recently been shown, where SSc exosomes induced an innate immune activation of keratinocytes, similar to that observed in SSc skin. RNA‐sequencing of exosome cargo highlighted lncRNA *H19* as a potential mediator of cell‐to‐cell communication in SSc.^4^ In the present study we confirm *H19* expression in SSc skin and show that specific manipulation of its expression can impact inflammatory, as well as fibrotic, phenotypes in skin. Specifically, we show for the first time that *H19* is up‐regulated in SSc skin and explanted dermal fibroblast compared with HCs. Importantly, we show that despite being variable, *H19* expression shows a moderate, albeit significant, positive correlation to well‐described profibrotic markers in SSc explanted dermal fibroblasts. In addition, despite the limited sample size, we observe an alignment between expression of *H19* and COL‐1, as well as STAT3, in SSc skin. This observation may offer a molecular basis to the known heterogeneity of skin fibrosis severity in SSc.^26^, ^27^

We identified *H19* as an epigenetic factor important for fibroblast activation. In vitro, knockdown of *H19* in SSc fibroblasts and overexpression of *H19* in healthy fibroblasts modulated profibrotic markers and the cell contraction ability of these cells. There are different regulatory mechanisms described downstream of *H19*, including sponging miRNA.^8^, ^35^ *H19* can bind different miRNAs such as miRNA17, miR29a, or miR196a.^22^, ^36^, ^37^, ^38^ Previous studies have shown downregulation of miR29a in SSc skin and fibroblast compared with HCs.^23^, ^24^ Moreover, reintroduction of miR29a in SSc fibroblast significantly reduced COL‐1 transcript levels.^24^ In addition, miR29a has been implicated in the regulation of STAT3 in cancer cells.^25^, ^26^ Because of our interest in understanding the role of *H19* in regulating profibrotic markers and hypothesizing a regulatory loop between STAT3 and *H19*, we further investigated the effect of miR29a in restoring *STAT3* and *Col1A1* levels in fibroblast overexpressing *H19*. Although this approach allowed us to better understand the regulatory role of *H19* in dermal fibroblast, it is important to consider that for the purpose of this study we did not analyze the basal levels of miRNAs in our *H19* overexpressing model. Moreover, the regulatory role of *H19* is complex and involves multiple mechanisms that could be explored in future studies.

**Figure 5:**
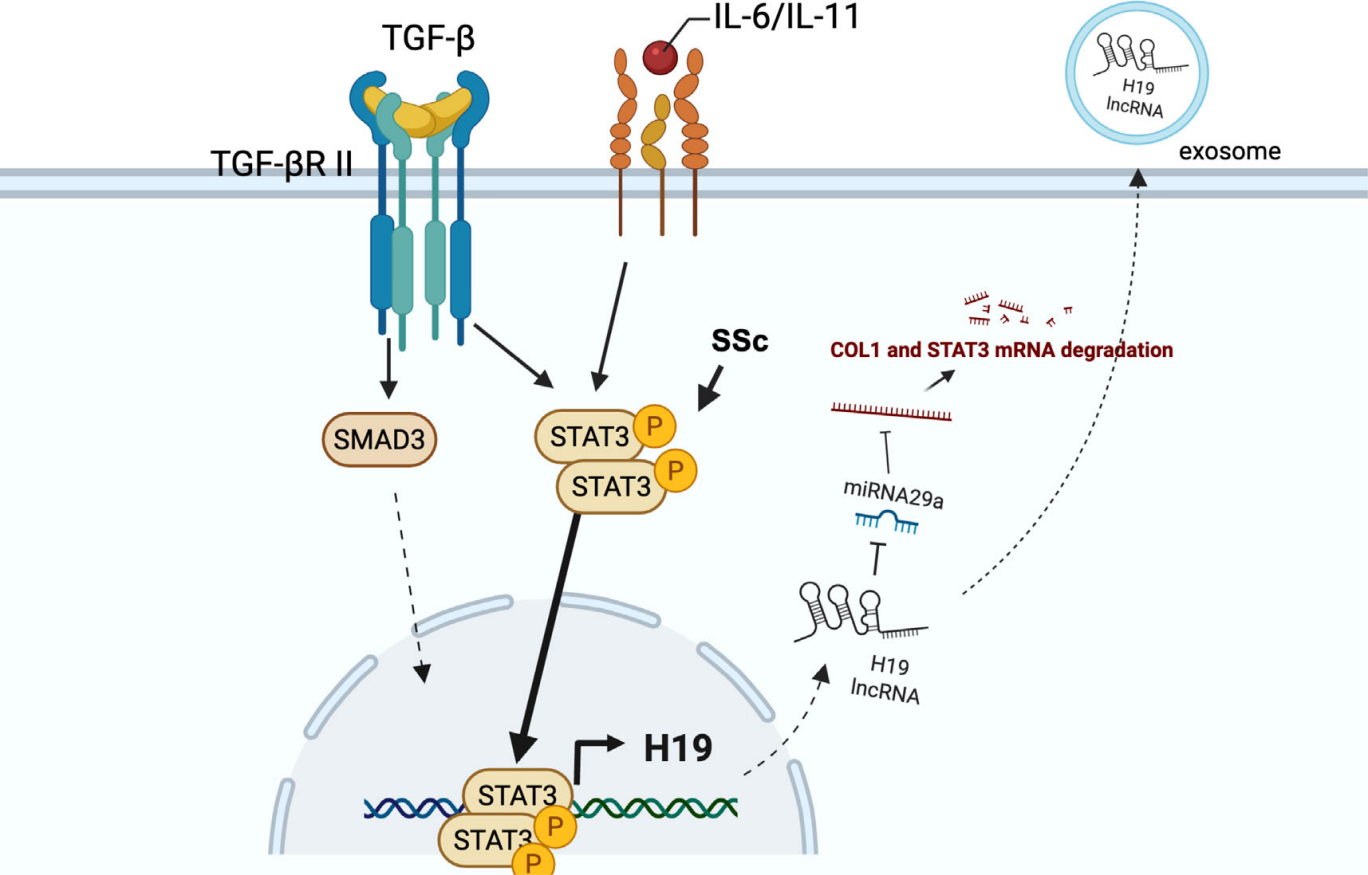
*H19* regulatory mechanisms in SSc fibroblast. TGF‐β and IL‐6/IL‐11, via SMAD3 and mainly STAT3 downstream signaling pathways, known to be increased in SSc, induce *H19* expression in dermal fibroblast. *H19* regulates profibrotic markers by sponging miRNA29a among other mechanisms described in the literature. We propose that *H19* is secreted into exosomes important in cell‐to‐cell communication. Created with BioRender.com. IL, interleukin; lncRNA, long noncoding RNA; miRNA29a, micro‐RNA 29a; mRNA, messenger RNA; SSc, systemic sclerosis; TGF‐β, transforming growth factor‐β. Color figure can be viewed in the online issue, which is available at http://onlinelibrary.wiley.com/doi/10.1002/art.70104/abstract.

Our study highlights that IL‐6, IL‐11, and TGF‐β, very well‐described factors in SSc, regulate *H19* expression mainly via the STAT3 signaling pathway (Figure 5), which has been shown to be instrumental for fibroblast to myofibroblast transition.^29^ In this context, additional studies to decipher the role of other imputed soluble mediators of profibrotic activation in SSc (such as Shh, NOTCH, and WNT) in the regulation of *H19* are warranted.

Our work supports the idea that aberrant *H19* expression in SSc dermal fibroblasts induces STAT3 and STAT1 activation in the adjacent keratinocytes (Figure 4). We propose that SSc fibroblasts secrete *H19* in exosomes as shown in our recent work.^4^ Although exosome analyses were beyond the scope of this study, our data showed that *H19* downregulation affects several signaling pathways. We therefore speculate that, alongside *H19*, other factors may play key roles in exosomal cargo composition and intercellular communication. It should be noted that exosomal *H19* may originate not only from fibroblasts but also from other cell types, including adipose mesenchymal stem cells and macrophages.^39^, ^40^

Although a complete characterization of inflammatory pathways fell outside the scope of this study, we found this particularly interesting as dysregulation of the type I interferon pathway and STAT3 have been implicated in the pathogenesis of SSc.^4^, ^29^, ^41^ Furthermore, a number of studies link *H19* to inflammatory pathways as NF‐κB, p38/MAPK/mammalian target of rapamycin, toll‐like receptor, and tumor necrosis factor‐α while this is context dependent and still unclear.^42^ One study suggested *H19* recruiting STAT1 into the nucleus to enhance the transcriptional expression of sorting nexin 10, promoting proliferation, invasion, and tube formation of fibroblast‐like synoviocytes in rheumatoid arthritis.^43^ Thus, a more detailed characterization of keratinocytes by transcriptomic analysis would be key to extend our analysis of *H19*'s role in skin inflammation in SSc. Interestingly, STAT3 can induce epithelial to mesenchymal transition (EMT), which supports a potential role of *H19* in the observed EMT in SSc skin. Inconsistencies about the role of *H19* and EMT are found in the literature showing either induction or reversion of EMT independently of EMT inducers.^42^ Further studies will be required to address this question.

Overall, our data shed new light into the complex pathogenesis of SSc and, together with previous works from our group, supports the notion of lncRNAs playing a key part in epigenetic regulation of SSc. Collectively, our study indicates that *H19* lncRNA is a key mediator of STAT3‐induced changes in SSc skin and could be considered a promising therapeutic target in tissue fibrosis.

## AUTHOR CONTRIBUTIONS

All authors contributed to at least one of the following manuscript preparation roles: conceptualization AND/OR methodology, software, investigation, formal analysis, data curation, visualization, and validation AND drafting or reviewing/editing the final draft. As corresponding author, Dr Del Galdo confirms that all authors have provided the final approval of the version to be published and takes responsibility for the affirmations regarding article submission (eg, not under consideration by another journal), the integrity of the data presented, and the statements regarding compliance with institutional review board/Declaration of Helsinki requirements.

## Supporting information


**Disclosure form**.

**Supplementary Table1: Demographics of SSc fibroblast cell lines (n=20)**.

**Supplementary Figure 1:** (A) RNAscope of *H19* (pink) in skin samples. Representative images of 2 HC and 2 SSc are shown. Left column represents epidermis area and right column deeper dermis of the same sample. Scale bar: 50 μm. (B) mRNA transcript levels of *H19* normalized to *GAPDH* mRNA levels in hTERT HC and SSc fibroblast. (C) RNAScope of *H19* (pink) co‐stained with COL‐1 (brown) in representative SSc skin sample with low *H19* levels. Scale bar: 50 μm.

**Supplementary Figure 2:** (A) Representative images of gel contraction assay from Figure 2C at 48h of SSc SCR and siRNA H19 1 fibroblast. (B) Area of collagen gel in percent (%) over time of SSc SCR (black) and *H19* knockdown fibroblast using siRNA 2 (grey). (C) mRNA transcript levels of *H19* in SSc fibroblast after 48h of gel contraction (96h after transfection). (D) Representative images of gel contraction assay from Figure 2H at 24h of Healthy control and H19OE fibroblast.
