# Supplementary material for: Long Noncoding RNA H19 Mediates STAT3‐Dependent Activation of Keratinocytes and Fibroblasts in Systemic Sclerosis Skin
Source: Arthritis Rheumatol. 2026 Apr 10;78(9):1929–41. doi: 10.1002/art.70104 (PMC13507581; doi:10.1002/art.70104)
Supplement: Supplementary file 1 [file ART-78-1929-s001.pdf]

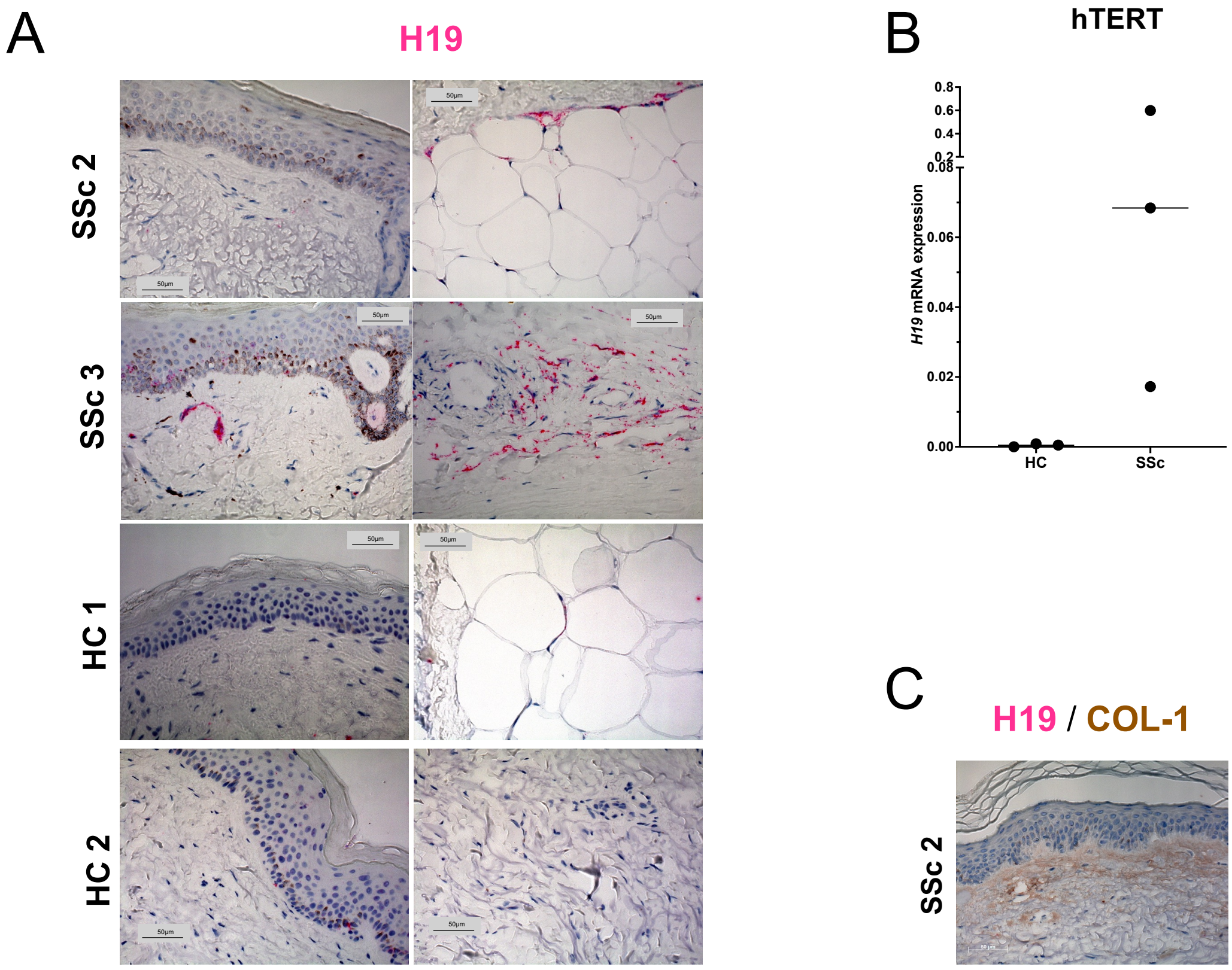
A
H19
SSc 2
SSc 3
HC 1
HC 2
B
hTERT
H19 mRNA expression
0.8
0.6
0.4
0.2
0.08
0.06
0.04
0.02
0.00
HC
SSc
C
H19 / COL-1
SSc 2
