# Supplementary material for: Long Noncoding RNA H19 Mediates STAT3‐Dependent Activation of Keratinocytes and Fibroblasts in Systemic Sclerosis Skin
Source: Arthritis Rheumatol. 2026 Apr 10;78(9):1929–41. doi: 10.1002/art.70104 (PMC13507581; doi:10.1002/art.70104)
Supplement: Supplementary file 2 [file ART-78-1929-s002.pdf]

ICMJE DISCLOSURE FORM

Date: 02/12/25____
Your Name: Begona Caballero-Ruiz____
Manuscript Title:__ Long Non-Coding RNA H19 mediates STAT3 dependent activation of keratinocytes and fibroblasts in Systemic Sclerosis skin ____
Manuscript number (if known):_ ar-25-1205.
____

In the interest of transparency, we ask you to disclose all relationships/activities/interests listed below that are related to the content of your manuscript. "Related" means any relation with for-profit or not-for-profit third parties whose interests may be affected by the content of the manuscript. Disclosure represents a commitment to transparency and does not necessarily indicate a bias. If you are in doubt about whether to list a relationship/activity/interest, it is preferable that you do so.

The following questions apply to the author's relationships/activities/interests as they relate to the current manuscript only.

The author's relationships/activities/interests should be defined broadly. For example, if your manuscript pertains to the epidemiology of hypertension, you should declare all relationships with manufacturers of antihypertensive medication, even if that medication is not mentioned in the manuscript.

In item #1 below, report all support for the work reported in this manuscript without time limit. For all other items, the time frame for disclosure is the past 36 months.

		**Name all entities with whom you have this relationship or indicate none (add rows as needed)**	**Specifications/Comments (e.g., if payments were made to you or to your institution)**
		**Time frame: Since the initial planning of the work**	
1	All support for the present manuscript (e.g., funding, provision of study materials, medical writing, article processing charges, etc.) **No time limit for this item.**	NIHR Biomedical Research Centre (NIHR BRC) (NIHR213331)	
		Susan Cheney endowment for Scleroderma Research	
		**Time frame: past 36 months**	
2			

	Grants or contracts from any entity (if not indicated in item #1 above).		
3	Royalties or licenses	_x___None	
4	Consulting fees	__x__None	
5	Payment or honoraria for lectures, presentations, speakers bureaus, manuscript writing or educational events	__x__None	
6	Payment for expert testimony	__x__None	
7	Support for attending meetings and/or travel	__x__None	
8	Patents planned, issued or pending	__x__None	
9	Participation on a Data Safety Monitoring Board or Advisory Board	_x___None	
10	Leadership or fiduciary role in other board, society, committee or advocacy group, paid or unpaid	_x___None	
11	Stock or stock options	__x__None	
12	Receipt of equipment, materials, drugs, medical writing, gifts or other services	__x__None	
13	Other financial or non-financial interests	__x__None	

Please place an "X" next to the following statement to indicate your agreement:

___X I certify that I have answered every question and have not altered the wording of any of the questions on this form.

ICMJE DISCLOSURE FORM

Date: 02/12/25___
Your Name: Christopher Wasson___
Manuscript Title:__ Long Non-Coding RNA H19 mediates STAT3 dependent activation of keratinocytes and fibroblasts in Systemic Sclerosis skin ___
Manuscript number (if known):ar-25-1205___

In the interest of transparency, we ask you to disclose all relationships/activities/interests listed below that are related to the content of your manuscript. "Related" means any relation with for-profit or not-for-profit third parties whose interests may be affected by the content of the manuscript. Disclosure represents a commitment to transparency and does not necessarily indicate a bias. If you are in doubt about whether to list a relationship/activity/interest, it is preferable that you do so.

The following questions apply to the author's relationships/activities/interests as they relate to the current manuscript only.

The author's relationships/activities/interests should be defined broadly. For example, if your manuscript pertains to the epidemiology of hypertension, you should declare all relationships with manufacturers of antihypertensive medication, even if that medication is not mentioned in the manuscript.

In item #1 below, report all support for the work reported in this manuscript without time limit. For all other items, the time frame for disclosure is the past 36 months.

		**Name all entities with whom you have this relationship or indicate none (add rows as needed)**	**Specifications/Comments (e.g., if payments were made to you or to your institution)**
		**Time frame: Since the initial planning of the work**	
1	All support for the present manuscript (e.g., funding, provision of study materials, medical writing, article processing charges, etc.) **No time limit for this item.**	NIHR Biomedical Research Centre (NIHR BRC) (NIHR213331)	
		Susan Cheney endowment for Scleroderma Research	
		**Time frame: past 36 months**	
2	Grants or contracts from any entity (if not indicated in item #1 above).		

3	Royalties or licenses	__x__None	
4	Consulting fees	__x__None	
5	Payment or honoraria for lectures, presentations, speakers bureaus, manuscript writing or educational events	__x__None	
6	Payment for expert testimony	__x__None	
7	Support for attending meetings and/or travel	__xx__None	
8	Patents planned, issued or pending	__x__None	
9	Participation on a Data Safety Monitoring Board or Advisory Board	__x__None	
10	Leadership or fiduciary role in other board, society, committee or advocacy group, paid or unpaid	__x__None	
11	Stock or stock options	__x__None	
12	Receipt of equipment, materials, drugs, medical writing, gifts or other services	__x__None	
13	Other financial or non-financial interests	_x___None	

Please place an "X" next to the following statement to indicate your agreement:

___X I certify that I have answered every question and have not altered the wording of any of the questions on this form.

ICMJE DISCLOSURE FORM

Date:_______________27.11.25____
Your Name:_Rebecca L Ross____
Manuscript Title:__ Long Non-Coding RNA H19 mediates STAT3 dependent activation of keratinocytes and fibroblasts in Systemic Sclerosis skin ____
Manuscript number (if known):ar-25-1205____

In the interest of transparency, we ask you to disclose all relationships/activities/interests listed below that are related to the content of your manuscript. "Related" means any relation with for-profit or not-for-profit third parties whose interests may be affected by the content of the manuscript. Disclosure represents a commitment to transparency and does not necessarily indicate a bias. If you are in doubt about whether to list a relationship/activity/interest, it is preferable that you do so.

The following questions apply to the author's relationships/activities/interests as they relate to the current manuscript only.

The author's relationships/activities/interests should be defined broadly. For example, if your manuscript pertains to the epidemiology of hypertension, you should declare all relationships with manufacturers of antihypertensive medication, even if that medication is not mentioned in the manuscript.

In item #1 below, report all support for the work reported in this manuscript without time limit. For all other items, the time frame for disclosure is the past 36 months.

		**Name all entities with whom you have this relationship or indicate none (add rows as needed)**	**Specifications/Comments (e.g., if payments were made to you or to your institution)**
		**Time frame: Since the initial planning of the work**	
1	All support for the present manuscript (e.g., funding, provision of study materials, medical writing, article processing charges, etc.) **No time limit for this item.**	NIHR Biomedical Research Centre (NIHR BRC) (NIHR213331)	
		Susan Cheney endowment for Scleroderma Research	
		**Time frame: past 36 months**	
2	Grants or contracts from any entity (if not indicated in item #1 above).	ASTRAZENECA	
		MITSUBISHI	
		KENNEDY TRUST	

3	Royalties or licenses	__x__None	
4	Consulting fees	__x__None	
5	Payment or honoraria for lectures, presentations, speakers bureaus, manuscript writing or educational events	__x__None	
6	Payment for expert testimony	_x___None	
7	Support for attending meetings and/or travel	__x__None	
8	Patents planned, issued or pending	__x__None	
9	Participation on a Data Safety Monitoring Board or Advisory Board	__x__None	
10	Leadership or fiduciary role in other board, society, committee or advocacy group, paid or unpaid	__x__None	
11	Stock or stock options	__x__None	
12	Receipt of equipment, materials, drugs, medical writing, gifts or other services	__x__None	
13	Other financial or non-financial interests	_x___None	

Please place an "X" next to the following statement to indicate your agreement:

___X I certify that I have answered every question and have not altered the wording of any of the questions on this form.

ICMJE DISCLOSURE FORM

Date:_______________27.11.25____
Your Name:_Francesco Del Galdo____
Manuscript Title:__ Long Non-Coding RNA H19 mediates STAT3 dependent activation of keratinocytes and fibroblasts in Systemic Sclerosis skin ____
Manuscript number (if known):ar-25-1205____

In the interest of transparency, we ask you to disclose all relationships/activities/interests listed below that are related to the content of your manuscript. "Related" means any relation with for-profit or not-for-profit third parties whose interests may be affected by the content of the manuscript. Disclosure represents a commitment to transparency and does not necessarily indicate a bias. If you are in doubt about whether to list a relationship/activity/interest, it is preferable that you do so.

The following questions apply to the author's relationships/activities/interests as they relate to the current manuscript only.

The author's relationships/activities/interests should be defined broadly. For example, if your manuscript pertains to the epidemiology of hypertension, you should declare all relationships with manufacturers of antihypertensive medication, even if that medication is not mentioned in the manuscript.

In item #1 below, report all support for the work reported in this manuscript without time limit. For all other items, the time frame for disclosure is the past 36 months.

		**Name all entities with whom you have this relationship or indicate none (add rows as needed)**	**Specifications/Comments (e.g., if payments were made to you or to your institution)**
		**Time frame: Since the initial planning of the work**	
1	All support for the present manuscript (e.g., funding, provision of study materials, medical writing, article processing charges, etc.) **No time limit for this item.**	NIHR Biomedical Research Centre (NIHR BRC) (NIHR213331)	
		Susan Cheney endowment for Scleroderma Research	
		**Time frame: past 36 months**	
2	Grants or contracts from any entity (if not indicated in item #1 above).	ASTRAZENECA	
		MITSUBISHI	
		KENNEDY TRUST	

3	Royalties or licenses	__x__None	
4	Consulting fees	Abbvie, Argenx, AstraZeneca, Boehringer-Ingelheim, Calluna, Chemomab, Certa, Cesas, DeepCure, Dyyalab, Engitix, Ergomed, Galderma, GlaxoSmithKline, IAG, Innovaderm, Kymera, Mitsubishi-Tanabe, Merck Sharp & Dohme, Mylteni, Novartis, Ono Pharmaceutical, Palisade, Qima, Quell, Serono, Syntara and Ventus	
5	Payment or honoraria for lectures, presentations, speakers bureaus, manuscript writing or educational events	__x__None	
6	Payment for expert testimony	__x__None	
7	Support for attending meetings and/or travel	__x__None	
8	Patents planned, issued or pending	_x___None	
9	Participation on a Data Safety Monitoring Board or Advisory Board	_x___None	
10	Leadership or fiduciary role in other board, society, committee or advocacy group, paid or unpaid	_x___None	
11	Stock or stock options	__x__None	
12	Receipt of equipment, materials, drugs, medical writing, gifts or other services	_x___None	
13	Other financial or non-financial interests	_x___None	

Please place an "X" next to the following statement to indicate your agreement:

___X I certify that I have answered every question and have not altered the wording of any of the questions on this form.
