# Supplementary material for: Long Noncoding RNA H19 Mediates STAT3‐Dependent Activation of Keratinocytes and Fibroblasts in Systemic Sclerosis Skin
Source: Arthritis Rheumatol. 2026 Apr 10;78(9):1929–41. doi: 10.1002/art.70104 (PMC13507581; doi:10.1002/art.70104)
Supplement: Supplementary file 3 [file ART-78-1929-s003.pdf]

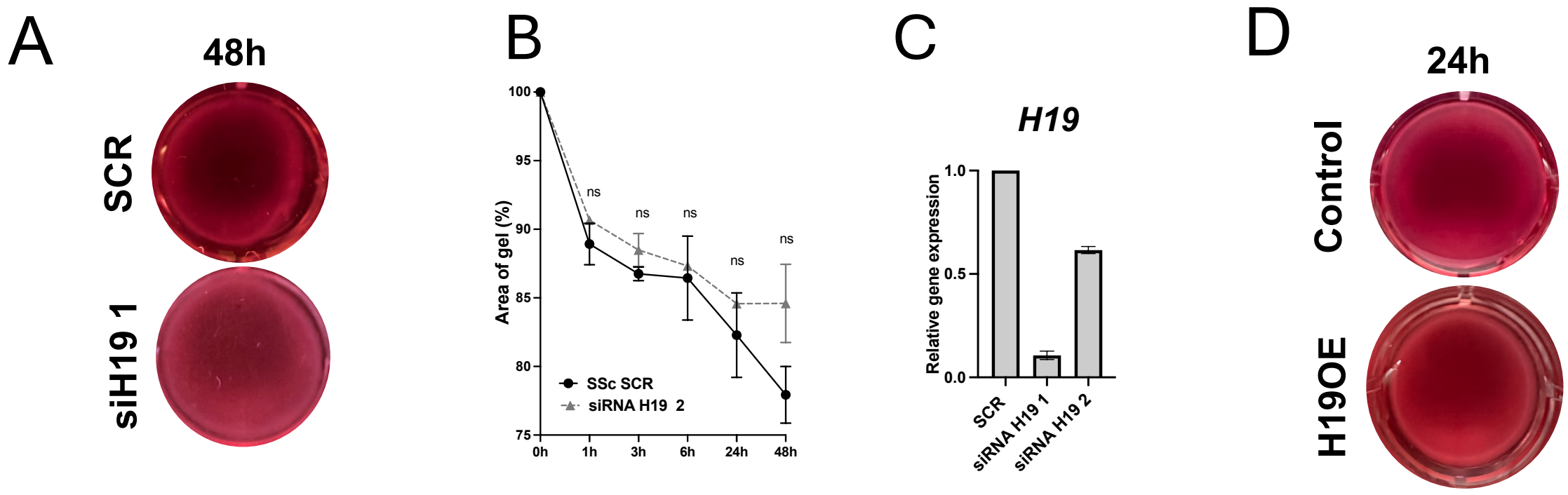
A
48h
SCR
siH19 1
B
Area of gel (%)
100
95
90
85
80
75
0h
1h
3h
6h
24h
48h
ns
ns
ns
ns
ns
SSc SCR
siRNA H19 2
C
H19
Relative gene expression
1.0
0.5
0.0
SCR
siRNA H19 1
siRNA H19 2
D
24h
Control
H19OE
