# Supplementary material for: Long Noncoding RNA H19 Mediates STAT3‐Dependent Activation of Keratinocytes and Fibroblasts in Systemic Sclerosis Skin
Source: Arthritis Rheumatol. 2026 Apr 10;78(9):1929–41. doi: 10.1002/art.70104 (PMC13507581; doi:10.1002/art.70104)
Supplement: Supplementary file 4 [file ART-78-1929-s004.pdf]

Supplementary Table 1: demographic and clinical features of dSSc patients

Patient information	dcSSc fibroblast cell lines (n=20)
**Age; mean ± SD**	50.2 ± 13.78
**Gender; %female**	55
**IS treatment; %MMF+**	65
**IS treatment; %MTX+**	25
**IS treatment; %Corticosteroid+**	40
**%ATA+**	40
**%RNA pol+**	25
**mRSS (total); mean ± SD**	25.7 ± 6.23

Age and mRSS expressed as mean ± standard deviation (SD). All the other characteristics shown as percentage (%). Abbreviations: IS, immunosuppressive therapy; MMF, mycophenolate mofetil; MTX, methotrexate; ATA, Anti-topoisomerase I antibodies (Scl-70); RNA pol, RNA polymerase III antibodies; mRSS, modified Rodnan skin score.
